# Supplementary material for: Are carbon emissions trading and green financial instruments synergistic? -Comprehensive quantitative research based on content analysis
Source: PLoS One. 2024 Mar 7;19(3):e0298601. doi: 10.1371/journal.pone.0298601 (PMC10919601; doi:10.1371/journal.pone.0298601)
Supplement: S1 Data — (ZIP) [file pone.0298601.s001.zip › Data/地级市Z_F工作报告.zip!/║■─╧/┬a╡╫╩╨/└┤╫╘╣1⁄2╓┌║┼í╛┬φ┐╦╩2╛▌═°í┐_2014.docx]

各位代表：

现在，我代表市人民政府，向大会作政府工作报告，请予审查，并请各位政协委员和列席人员提出意见。

一、2013年工作回顾

2013年，是宏观环境异常复杂、经济形势极为严峻的一年，经济下行和结构调整双重压力给地方发展带来巨大挑战。我市作为老工业基地、资源型城市、欠发达地区，受到的影响首当其冲、更加直接。面对前所未有的困难局面，全市上下按照省委、省政府统一部署，在市委的坚强领导下，在市人大、市政协的监督支持下，团结奋进，克难攻坚，强力推进六个“四年行动计划”，科学应对历史罕见旱情，有力确保了全市经济社会平稳较快发展。

（一）市域经济在加速赶超中取得新进展

经济实力稳步提升。2013年，全市完成地区生产总值1115亿元（预计数，下同），增长10.5%，其中第一、二、三产业分别实现增加值163亿元、603亿元、349亿元，增长2.5%、10.9%、13%。完成公共财政总收入103.9亿元，增长17.28%，增速居全省第4，其中地方公共财政预算收入62.41亿元，增长20.42%，增速居全省第3，均为历史最好成绩。完成固定资产投资786.5亿元，增长35.2%。实现社会消费品零售总额338.5亿元，增长13.7%。实现进出口总额14.5亿美元。城镇居民人均可支配收入达到16300元，增长8.8%；农村居民人均可支配收入达到6550元，增长12.8%。

发展后劲显著增强。经济结构不断优化，三次产业结构由2012年的14.9:55.1:30调整为14.6:54.1:31.3，第三产业占比提高1.3个百分点。项目建设有力推进，全年施工项目1780个，其中新开工项目1281个，竣工项目800个，开发储备重点项目573个，争取国、省项目补助资金达97.1亿元。园区平台更加夯实，全市园区建成面积达到38平方公里，入园企业448户，完成园区投资185亿元，建设标准化厂房140万平方米。政策争取积极有效，我市成功纳入全国老工业基地调整改造规划和资源型城市可持续发展规划，列入全国循环经济示范城市、全国二级物流园区布局城市，发展机遇更加利好，政策支持力度更大。

顶层设计更为科学。着眼于推进城乡统筹、区域融合、城市空间拓展和产业布局优化，广泛征求意见，汇聚各方智慧，形成了推进市域可持续发展的“融城战略”，确定了建设“两大经济板块”，打造“两条产业走廊”，推进我市与长株潭地区，市内相邻县、市、区，以及县（市、区）城区与周边乡镇融合发展的总布局，并先期启动了贯穿五县市区的娄底大道建设，为促进市域融合发展、建设幸福娄底、实现全面小康规划了崭新的路线图。

（二）产业发展在转型升级中迈出新步伐

服务业量质齐升。强力推进服务业发展四年行动计划，完成第三产业投资397亿元，占全市总投资的51%，高于全市投资增速14.1个百分点。其中46个商贸物流旅游重大项目完成投资59.4亿元，大物流、大商贸格局初具雏形。文化旅游业加快发展，一批旅游基础设施相继竣工，配套建设加速推进，接待能力大幅提升，大梅山文化旅游年暨“万辆自驾游”等一批促销活动成功举办，极大提升了娄底的美誉度和知名度，全年旅游总收入突破百亿元大关。新化县跻身全省文化旅游特色产业重点县，双峰县成功创建全省旅游强县，湄江国家地质公园成功创建4A级景区。房地产、住宿餐饮、批发零售等传统服务业和金融、会展、信息、中介、咨询、健康养老等新兴服务业蓬勃发展。

工业结构不断优化。扎实推进工业发展四年倍增计划和工业企业（项目）四年帮扶行动，全年实现规模工业增加值424亿元，增长10.6%，其中高技术产业增长16.2%，高于规模工业增速5.6个百分点，六大高耗能行业占规模工业的比重下降6.1个百分点。战略性新兴产业实现增加值54亿元，增长12.6%。完成工业技改投资226亿元，信息化建设投资3.4亿元，分别增长1.4%、48%。项目对接卓有成效，全市新开工工业项目337个，竣工投产53个，汽车板项目加速推进，完成投资17.6亿元，有望今年6月投产。

现代农业加速发展。扎实开展抗旱救灾、森林防火和动物防疫等工作，农业生产稳中有增。全年播种粮食407.57万亩，总产157.5万吨。农业现代化加速推进，流转耕地41.35万亩，推广新机具1.78万台（套），农民专业合作社增加到1034个。新增农产品加工企业41家，总数达到1126家，实现销售收入117亿元，增长23.1%；新增亿元以上企业8家、市级龙头企业25家，国家级、省级、市级龙头企业分别达到1家、20家、141家。632家休闲农业企业完成营业收入15亿元，增长20.2%。“三黑一菌”等特色产业快速发展。

（三）城乡面貌在统筹发展中展现新变化

环境整治取得突破。全力推进市区一体、全民创卫，创建国家卫生城市取得阶段性重大成果，顺利通过国家评估，娄星区被推荐为全省城乡环境卫生十佳县市区。以创国卫为引领，举全市之力深入推进城乡环境整治与建设四年行动计划，一批交通、市政、环卫等基础设施相继完善，公共交通建设和交通秩序管理不断加强，城中村、城乡结合部得到有效整治，城乡环境卫生全面改善，极大地增进了民生福祉，凝聚了人心士气，密切了干群关系，极大地激发了全市人民改善人居环境、共创美好家园的热情。

交通条件显著改善。务实推进交通建设四年行动计划，全年完成投资41.8亿元。沪昆客运专线、娄邵铁路扩能改造工程进入铺轨阶段，新溆、安邵、长韶娄等3条高速和娄北连接线等项目加快推进。经过各级人大代表、政协委员积极建言，益娄、娄衡高速实现实质性开工。国省干线公路改造完成投资5.3亿元，新（改）建里程65.7公里。中心城区和县（市、区）城区路网加速完善，新增城市道路里程10.6公里，农村公路里程559公里，城乡居民出行更加便捷。

城镇建设加速推进。全年共开工建设城建项目128个，完成投资87亿元。其中娄底中心城区开工建设项目91个，完成投资59.57亿元，孙水河南岸风光带、华达地下通道等重点城建项目进展顺利。加快县市城区扩容提质，双峰城北新区、涟源城东新城、冷水江城东生态城、新化北塔综合区加快建设。小城镇建设亮点纷呈，涌现出了杉山－小碧－双江、岩口－铎山－渣渡、杨市－枫坪－茅塘、青树坪－三塘铺－甘棠等一批小城镇群，以及荷叶、水车等一批产业特色镇。

农村基础不断夯实。全面实施绿化娄底四年行动计划和绿色娄底建设，造林16.67万亩，“三边一区”绿化成效显著。划定永久基本农田219.5万亩，申报土地整理16.04万亩，落实娄邵盆地基本农田建设第一期投资2.01亿元。投入水利资金8.48亿元，除险加固小Ⅱ型水库165座，治理河道17公里，解决23.15万农村人口饮水安全问题。5个省委、省政府为民办实事新农村示范村建设加快推进，完成投资1.51亿元。

（四）社会事业在协调推进中有了新进步

创新发展能力加强。全市荣获省级科技进步奖4项，专利授权713件，高新技术企业达到60家，其中国家重点火炬高新技术企业4家。我市首家国家级实验室——国家电子陶瓷产品质量监督检验中心投入使用。“锑清洁冶炼关键技术研究与示范”获得省科技重大专项支持。我市荣获全国科技进步先进市称号，涟源市被评为全国科普先进市。

民生改善力度加大。尽心竭力为民办实事，全年完成各项民生支出121.5亿元，占公共财政预算支出的64.36%。新增城镇就业人员4.03万人，城镇登记失业率控制在4.3%以下，新增农村劳动力转移就业6.7万人。城镇基本社会保障制度日趋完善，新型农村社会养老保险和城镇居民养老保险实现全覆盖。城、乡低保月人均救助水平分别达到257元和118元，农村五保分散和集中供养对象年人均供养标准分别提高到2400元和4800元。新建和改扩建敬老院15所。发放医疗救助资金6000万元、临时救助资金1000万元，分别救助22万人次和2.6万户次。公益慈善事业蓬勃发展，募集基金4700万元，志愿者达到1.1万人。推行乡镇卫生院住院费用起付线外全报销制度，新型农村合作医疗参合率达99.38%。新开工保障性住房2.61万套，基本建成2万套，改造农村危房6170户。加强住房公积金保障，发放贷款8.7亿元。投入各类扶贫资金7.6亿元，减少贫困人口20.9万。发放移民后扶直补资金1.21亿元，安排项目资金4.37亿元。建设老区帮扶项目93个，完成投资3224万元。着力推进“菜篮子”三年行动计划，在全省率先建成9家平价菜市。

各项事业协调推进。全力以赴推进化解大班额四年行动计划，全年完成投资4.5亿元，启动建设项目45个，竣工18个，交付学位1.56万个，中心城区小学平均班额降至65人左右。建设农村义务教育合格学校155所，维修改造中小学校舍152所。加强学生营养餐规范管理，切实落实整改措施，新建学校食堂219个，改造222个。全面推行基本药物零差率销售，基层公立医疗机构实现基本药物制度全覆盖。冷水江市公立医院改革试点稳步推进。市中心医院新住院大楼以及一批县市区医院改扩建项目建成使用。市文化中心建设有序推进，农村书屋实现村村覆盖，新化北塔、红二军团长征司令部旧址和双峰光甲堂成功获批全国重点文物保护单位。第十二届省运会筹备工作扎实有序，场馆建设顺利推进。人口计生综合改革不断深化，人口自然增长率稳定在7‰以内。

社会大局和谐稳定。“天网工程”全面启动，中心城区55条主要街道楼牌门牌设置顺利完成，扫黑除恶、扫黄禁赌禁毒等专项治理成效明显，平安娄底建设扎实推进，社会管理综合治理民调排名全省第七，比上年前移6个位次。企业安全标准化、煤矿“四化”和瓦斯治理攻坚强力推进，隐患排查与专项治理力度不断加大，生产安全、道路交通安全总体平稳。严厉打击非法集资，积极做好金融风险防控和处置预案，金融安全区和“省金融生态良好城市”创建深入推进。应急处置能力不断提高，成功处置冷水江禾青森林火灾等突发事件。食品药品专项整治不断加强，监管能力显著提升，娄星区成功创建首批国家餐饮食品安全示范区。“12345”市长热线正式开通，群众诉求渠道不断畅通，人民调解不断强化，信访积案有效化解。

（五）改革开放在强力攻坚中呈现新气象

两型发展稳步推进。进一步理顺体制机制，科学划分六大两型示范功能分区，分解落实了示范区考核指标体系。冷水江市、双峰县经开区分别获批全省循环经济示范县（市）、示范园区。“九大环保工程”29个项目全面完成，实现总投资14.73亿元，重点流域、锡矿山地区环境污染综合治理初见成效。61个减排项目全部建成运行，水泥和火电行业脱硫、脱硝工程全面完成。中心城区饮用水源水质达标率100%、环境空气质量优良率达到93.83%。

要素瓶颈有效破解。强力推进“营改增”改革，深化与涟钢等重点企业对接，切实涵养了税源，保障了财政支出。加强政银企对接，推进债券融资，积极引进金融机构，全市存款余额达到1038亿元，增长15%；贷款余额达到638亿元，增长17%；贷存比排名全省第四。强化规划管控，统筹土地报批，全年共报批用地项目163个，总用地2.3万多亩，增长17%，清理闲置用地2730亩。推进征拆安置创新，严格控违拆违，有效保障了项目的顺利推进。

开放水平不断提高。大力实施“娄商回娄”战略，第三届娄商大会签约项目38个、资金201.7亿元，娄商广场开工建设。“湘博会”晋级为省政府主办节会，第四届“湘博会”签约项目250个、资金272亿元。有效利用“中博会”、“港洽周”等招商平台，切实提高招商引资实效，全年内联引资305亿元，实际到位外资2.81亿美元，分别增长41.2%、19%。加强区域合作，与广东省佛山市结为友好城市。加快实施“走出去”战略，新增境外投资企业5家。着力推进口岸建设，已先期开展海关等业务。

（六）政府效能在发展实践中得到新提升

认真落实市人大及其常委会的决议决定，主动接受人大法律和工作监督、政协民主监督和社会各界的监督，办结人大代表议案1件、建议229件，政协委员提案188件，解决了一批百姓关注的热点、难点问题。加强法治政府建设，严格执行重大行政决策公众参与、专家论证、合法性审查、风险评估、集体讨论和责任追究制度，在全省率先出台《行政执法监督办法》。强力推进建设项目并联审批和“两集中两到位”改革，大力精简行政审批项目，全面推进电子政务，深化政务公开，行政效能、服务质量有效提升。持续开展作风建设活动，明查暗访、集中督查66次，处理违纪人员115人。深入开展“发展环境优化年”活动，严厉打击“三强”、“两突”行为，发展环境有所改善。进一步清理和规范市直行政事业单位国有资产，严格按要求停止楼堂馆所建设。加强审计监督，完成政府投资项目结算审计330个，核减资金2.86亿元。

国防动员和后备力量建设扎实推进，全民国防意识明显加强，征兵工作调整改革顺利进行，驻娄解放军、公安、消防、武警履行使命能力持续提升，特别是在处置疑难险重任务中敢于担当、不怕牺牲，为全市经济社会发展作出了突出贡献。第三次经济普查全面启动，统计基础作用和监测服务水平不断提升。物价、民族宗教、人防、对台、外事侨务、档案、保密、残联、地震、仲裁、工会、科协、红十字会、共青团、妇女、儿童、老龄等各项工作都取得了新进步。

各位代表，过去一年成绩的取得，是市委坚强领导和市人大、市政协有力监督支持的结果，是全市广大干部群众心血和汗水的结晶，也离不开社会各界人士的大力关心和支持。在此，我代表市人民政府，向全体人大代表、政协委员和全市人民，向各民主党派、工商联、无党派人士，向驻娄部队指战员、武警、消防官兵、政法干警，向中央、省属单位的各位朋友，向所有关心、支持娄底建设和发展的同志们、朋友们，致以崇高的敬意和衷心的感谢！

成绩来之不易，困难前所未有，问题不容忽视。尽管我们付出了很大努力，但经济社会发展中仍存在一些困难和问题：因宏观形势趋紧，发展速度总体放缓，经报请市人大常委会批准，对部分指标进行了调整。同时，产业结构仍然不优，转型发展任务繁重；关键领域改革难以突破，要素“瓶颈”制约仍然明显；财政收支矛盾加大，民生改善任重道远；生产、生态、金融三大安全问题压力仍然很大；政府职能亟待转变，发展环境不优问题仍然突出，等等。我们将高度重视，努力加以解决。

二、2014年工作安排

2014年，是贯彻落实党的十八届三中全会精神、全面深化改革的第一年，也是实施“十二五”规划和推进六个“四年行动计划”的关键之年。国际国内形势依然复杂严峻，世界经济深度调整，国内经济进入中速增长期，稳增长、调结构的压力很大。但同时也应看到，我国经济发展长期向好的基本面没有改变，特别是党的十八届三中全会对全面深化改革作出了总体部署，将进一步激发发展的内生动力和活力。只要我们保持清醒头脑，牢牢把握工作主动权，增强忧患意识，坚持底线思维，抢抓发展机遇，搞好统筹兼顾，就一定能够赢得发展新优势、开创发展新局面、实现发展新跨越。

2014年政府工作的总体思路是：坚持以邓小平理论、“三个代表”重要思想和科学发展观为指导，全面贯彻落实党的十八大、十八届三中全会以及中央、省、市经济工作会议精神，继续坚持稳中求进的工作总基调，把改革创新贯穿于经济社会发展各个领域各个环节，坚定实施“科学发展，加速赶超”战略，强力推进六个“四年行动计划”，促进经济社会协调、安全、高效、可持续发展，为加快建设幸福娄底、全面建成小康社会奠定更加坚实的基础。

2014年的主要预期目标是：地区生产总值增长10.5%以上，其中第一、二、三产业分别增长2.5%、11%、13%以上；规模以上工业增加值增长11%以上；固定资产投资增长25%以上；公共财政总收入、地方公共财政预算收入均增长12.5%以上；社会消费品零售总额增长13.5%以上；城镇居民、农村居民人均可支配收入分别增长11%、12%以上；人口自然增长率、城镇登记失业率分别控制在8.8‰、4.3%以内；节能减排完成省定目标。

围绕目标要求，着力抓好五个方面工作。

（一）以改革创新为突破，增强发展动力

大力推进重点领域改革。不折不扣贯彻中央关于全面深化改革的总体部署，确保各项改革举措落到实处、取得实效。紧紧围绕使市场在资源配置中起决定性作用深化经济体制改革，努力降低市场准入门槛，营造公平有序的政策环境、市场环境和社会环境。深化金融领域改革，加快推进企业产权多元化和资产证券化，充分利用好股票发行注册制改革和湖南股交所推出“培育板”的契机，支持企业上市融资，提高直接融资比重；加强融资平台建设，逐步实行城投投建分离；加大金融支持经济结构调整和转型升级的力度，进一步强化政银企合作和融资担保服务，推动多元化金融组织体系建设，引导更多资金投向实体经济，为企业尤其是中小微企业发展提供有力支撑。深入推进财税体制改革，严格债务风险控制和税收管理，整合专项资金，原则上不设立新的专项。鼓励、支持、引导非公有制经济发展，扶持有一定规模的个体户注册为企业，帮助符合条件的私营企业转型升级。加快建立城乡统一的建设用地市场，探索农村集体经营性建设用地出让、租赁、入股，实行与国有土地同等入市、同权同价。积极推进农村综合改革，深化农业经营体制、土地管理制度、农村金融、水利综合等相关改革，破解制约现代农业发展的体制障碍。深化医药卫生体制改革，落实国家基本药物制度，加快公立医院、基层医疗卫生机构综合改革。深化机关事业单位人事制度改革，统筹推进各类人才队伍建设。

大力推进创新发展。依靠科技创新和技术进步激活内生动力，推动集约节约、转型升级、科学发展。以产业政策为导向，加快组建“湖南省锑业工程技术研究中心”，提升涟钢企业技术中心创新能力，建设双峰农机机电技术服务平台。以重点行业为突破，主攻新材料、机械制造、节能降耗等领域，攻克一批支撑优势产业发展的关键技术，推动产业高端化。以项目培育为抓手，把创新驱动发展战略深度融合到具体产业、项目和产品上来，把先进科技、优秀人才汇聚到大项目、好项目上来。支持企业平台建设、自主创新研发，不断提高自主创新能力。

大力推进开放合作。强化招商引资规划和管理，全面落实“娄商回娄”战略，加强签约项目的落地开工，加快娄商广场建设，大力发展总部经济。加强与佛山市的友好合作，抓实不锈钢等产业的对接，提高招商引资实效。积极对接世界500强、央企、优质民营企业，加强市企合作，以资源引资金、以老厂引老板、以内商引外商，努力引进一批大企业、好项目和战略投资者。办好第九届湖南西部农博会。确保全年内联引资增长25%以上、实际到位外资增长15%以上。着力调优外贸结构，支持进出口企业做大做强，积极拓展对外经济合作，鼓励引导企业“走出去”，大力推进服务外包基地建设，发展服务外包产业，促进外派劳务输出。确保全年实现进出口总额16亿美元，增长10%以上。

（二）以全面小康为目标，切实改善民生

努力增加居民收入。坚持分类指导，加快全面建成小康社会进程。充分利用好武陵山片区区域发展与扶贫攻坚政策，加大扶贫攻坚力度，大力实施精准扶贫，确保减少贫困人口17.8万。把就业摆在更加突出的位置，抓好省级创业型城市建设，重点扶持大学生、农民工等群体创业，加大对离校未就业高校毕业生的就业援助力度，完成农民素质教育和技能培训20万人次以上，新增城镇就业3.4万人以上，新增农村劳动力转移就业5万人以上，增加农民创业和就业收入。扶持发展联户经营、专业大户和家庭农场，扩大农民财产性和经营性收入。坚决落实各项支农惠农政策，让每一位农民得到实实在在的好处，努力增加农民政策性收入。

协调发展社会事业。坚持教育优先发展，加大教育投入。继续推进化解大班额，确保全年新增1.68万个学位。切实抓好校车安全工作，推进校安工程、平安校园建设。加强教师队伍建设，促进义务教育均衡发展，努力让每一个学生都享有公平受教育机会，进一步提高学生综合素质。大力发展文化事业和文化产业，完成文化中心主体工程，加快乡镇文化站、农家书屋、职工书屋、群众文化活动示范点、广播村村响、电视村村通建设，推进“三馆一站”免费开放。加快重点医疗卫生项目和基层医疗卫生设施建设，着力提高医疗服务水平，逐步实现公共医疗卫生服务均等化。全力做好第十二届省运会组织筹备工作，全面完善比赛场所和配套设施建设，确保圆满成功。

着力完善社会保障。优化财政支出结构，集中财力保重点保民生，确保民生支出比重达到60%以上。建立资源共享的社会养老保险信息平台，促进养老服务社会化，推动城乡养老、失业、医疗、生育、工伤保险全覆盖。完善新型农村合作医疗制度，切实提高大病保障水平。建成各类保障性住房3.19万套，加快推进住房保障和供应体系建设，满足群众基本住房需求。完善防灾减灾救灾、应急预案和救援体系，加强低收入、困难群体救助救济，切实维护弱势群体合法权益。加大移民后扶力度，扎实推进特困移民解困避险工程；抓好“两项制度”有效衔接工作，帮扶贫困人口4.55万。进一步加强“菜篮子”工程建设，强化价格调控和产品质量监管，保持市场、物价稳定，保障群众基本生活需要。

全面创新社会治理。抓实生产安全，落实“党政同责、一岗双责、齐抓共管”要求，强化企业主体责任，有效遏制各类安全事故发生。强化质量监督和工商管理，加强卫生监督和食品药品监管，确保公共卫生和食品药品安全。全力推进金融生态良好城市建设，严厉打击非法集资，有序规范民间融资，切实防控和规避金融风险。抓好人口计生工作，切实提高人口素质，有序推进单独二孩政策落实，稳定低生育水平。加快“天网工程”建设，加强社区建设管理，推进综合执法，维护司法公正，畅通信访渠道，化解社会矛盾，着力打造平安娄底。

（三）以转型发展为引领，推进产业升级

推进服务业现代化多元化。加快现代商贸物流发展，立足“一核二区四节点”整体布局，重点推进新合作湘中国际物流园、湖南诚通现代物流园等大型物流项目建设，着力完善城市商圈和便利店建设，继续实施新农村现代流通服务网络建设工程。规范和整顿市场秩序，优化消费环境，激发消费活力。促进文化与旅游深度融合，以“湖湘文化体验区、梅山文化生态区、中心城市休闲区”为框架，以“一点一线”为重点，着力培育“乡间侯府、梯田王国、山水围城、世界锑都”等旅游品牌，加快打造“湘中明珠，快乐娄底”新兴旅游目的地；启动创建紫鹊界梯田和曾国藩故里5A级景区、大熊山4A级景区；大力发展农业休闲旅游，重点建设一批生态旅游休闲小镇和历史文化名镇名村。确保全年旅游接待人次、旅游总收入均增长20%以上。提升现代金融业，大力引进和培育商业银行、村镇银行、民资银行，完成农信社改制组建，加快市金融产业园建设。规范和加强物业管理，推动房地产业健康有序发展。推进“数字娄底”建设，加快发展信息、咨询、电子商务、健康养老、家政服务等新兴业态。

推进工业新型化集群化。以转型升级为目标，以“四转三化”为路径，强力推进工业化和信息化深度融合，促进工业转型升级，提升企业竞争力。加快改造传统产业，大力支持涟钢、冷钢、闪星锑业等骨干企业转型发展、做大做强，引导钢铁、煤炭、有色等资源型产业向精深加工和链式循环发展；继续整合煤炭资源，加快组建煤业集团步伐，大力发展煤化工产业，促进煤炭产业转型升级。加快发展战略性新兴产业，重点培育工程机械、汽车零部件等先进装备制造和新能源、新材料、电子信息、医药食品、节能环保等产业，抓好汽车板、不锈钢等重大项目建设，支持一批高新技术企业做大做强。加快提升特色产业，加大农机、煤机、电子陶瓷、高级硅砖等特色产业整合力度，组建大型企业集团，打造特色产业基地。全力推进以娄底经济技术开发区为龙头的各级各类园区建设，坚持工商并举、产城一体，促进要素向园区集聚、产业向园区集中、土地在园区集约，把园区打造为产业转型的主阵地。

推进农业产业化特色化。抓好新增粮食产能工程、80亿粮油产业工程、娄邵盆地基本农田建设等项目，加强粮食生产，确保全年粮食播种面积400万亩以上，粮食总产稳定在160万吨左右。加快构建新型农业产业化经营体系，推进农村土地依法有序流转，引导农民合作社、种养大户、家庭农场等新型经营主体整合发展，力争新增亿元农产品加工企业5家以上。强力推进农业标准化建设，大力发展无公害农产品、有机农产品、绿色食品和地理标志产品。加快发展农业特色产业，大力支持“三黑一菌”特色品牌做大做强。提高农业耕种收机械化水平，实施水利建设“866”计划，加快推进娄邵干旱走廊综合治理，抓好耕地保护、防汛抗旱、动物防疫、森林防火等基础工作，增强农业综合生产与保障能力。

（四）以融城战略为总揽，统筹城乡发展

加快推进融城步伐。切实推进以人为核心的新型城镇化，根据资源环境承载能力构建科学合理的城镇化宏观布局。加快编制全市融城发展总体规划，加强娄底大道沿线小城镇的规划建设，逐步建立市区一体、上下联动、共建共享的融城推进机制。大力推动交通融城，加快沪昆客运专线、娄邵铁路和新溆、安邵、长韶娄、益娄、娄衡等高速公路建设，努力争取湄琅高速年内开工，加快娄底大道、城市环线、高速连接线、站场设施等城乡路网建设，进一步加大干线公路改造力度，加快县（市、区）及重点镇快速通道和重点景区旅游通道建设，拉近心理距离，构建交通融城骨架。大力推动产业融城，立足现有产业基础和资源优势，坚持错位发展，优势互补，加强产业协同，优化娄底大道沿线产业的规划布局，夯实融城基础，增强融城实力。加强主要干道两厢和重点乡镇土地控制与开发，依法严厉打击违法违规乱建抢建行为。大力推动文化融城，加强文明创建、公民道德与社会主义核心价值体系建设，进一步提升市民素质和文化软实力、竞争力。强化“坚韧、勤奋、尚德、自强”的娄底精神，增强娄底意识，进一步提高全市人民作为娄底人的认同感、归属感、自豪感。

扎实推进两型建设。积极争取全市整体纳入湘江流域生态文明先行示范区范围，全面开展两型示范创建活动，建设一批两型特色明显的示范点和综合示范区域。加快两型示范带动，按照打造娄底城市新形象的标准，重点推进万宝新区、东部新区两型示范建设。加强资源节约利用，实施十大重点节能工程，推行合同能源管理，抓好产业废物综合利用示范基地、再生资源产业园等项目，以循环经济示范县、示范园区和示范企业建设为重点，稳步推进国家循环经济示范城市建设。加强生态文明建设，全面启动水环境综合治理，实行最严格的水资源管理制度，加大仙女寨－水府庙生态公园保护开发和矿山植被修复治理力度，争取把柘溪库区纳入全省、全国生态环境保护规划，努力把锡矿山地区建设成全国资源型城市“两型社会”建设生态文明示范区和重金属污染治理样板区。

着力推进城乡一体化。按照新型城镇化要求，围绕投资拉动明显、保障城镇安全、改善社会民生等重点领域，加快城镇基础设施转型升级，全面提升城镇综合承载力。进一步巩固创卫成果，建立健全市级统筹、城乡统一的环境管理监测体系，让天更蓝、山更绿、水更清。加大城乡公共基础设施投入和建设力度，重点抓好饮水安全、天然气入户、农村水电改造等工程建设，启动涟水河、孙水河风光带规划。加强新农村建设的规划、指导，切实加强以保畅保洁为重点的农村公路和干线公路养护，加快城市公共服务向农村延伸，建设富裕、生态、宜居、平安、活力新农村。加大对冷水江市统筹城乡发展、整体推进新农村建设试点支持力度，以点带面推动全市城乡一体化步伐。坚持保护与开发并重，用心保留历史文化遗存和传统公共空间，让远离家乡的游子想得起童年往事，记得住浓浓乡情，产生出深深眷恋。

（五）以项目建设为抓手，扩大有效投资

进一步抓实项目前期。把准国家产业政策、投资导向和重点，将已争取到的各类利好政策落实到具体项目上，搞好上下对接，力争更多的项目和资金落户我市，确保开发储备重大项目200个以上。做细做深做实项目前期，重点抓实薄板深加工产业园、不锈钢产业基地、安张衡铁路、全国慈善文化产业园、报业集团等一批事关产业转型、基础配套、民生改善的重大项目前期工作，确保项目“报得上、批得下、落得地”。

进一步抓实项目调度。重点围绕“四个一批”项目，强化分析调度。以项目落地为原则，明确责任主体，加强对已签约项目的跟踪落实，创造条件推动项目开工建设。以项目竣工投产达效为目标，着力协调解决审批、土地、资金、环境等具体问题，促进项目尽快建成投产。确保2014年项目投资总规模达到380亿元左右，其中工业项目完成投资113.7亿元；交通建设项目完成投资58.6亿元；城建项目完成投资77.8亿元；农业农村项目完成投资33.3亿元；商贸物流旅游项目完成投资65.7亿元；社会事业项目完成投资30.8亿元。

进一步抓实项目保障。创新投融资模式，充分发挥市场机制作用，吸引和鼓励社会资本参与基础设施和基础产业建设。强化重点项目“一对一”融资，深化政银企合作，加大对全市项目建设、经济增长和民生改善的融资力度。切实加强国土经济运行调度，合理确定基准地价，努力提高供地率，积极探索市城建投与土地储备中心的深度融合，逐步建立市级统筹、兼顾各方的土地融资体系。注重土地综合开发利用，加强土地储备与项目合理配置，做活土地经济文章。继续加大“三强”、“两突”打击力度，进一步优化征地拆迁和项目施工环境。

与此同时，紧紧围绕创建“双拥模范城”目标，大力推进与国家发展战略相顺应、与娄底经济社会发展相互动、与履行职能使命相对称的国防动员和后备力量建设，推动军民融合深度发展。切实抓实统计基础工作，抓好第三次经济普查和全面小康监测考核工作，提高统计监测评估和参谋服务水平。加快推进市档案馆建设。认真做好第九次村委会换届工作，切实关心关爱基层干部。统筹抓好国资监管、民族宗教、港澳台、外事侨务、人防、科协、地方志、住房公积金、保密、气象、水文、仲裁、慈善、残联和工会、红十字会、共青团、妇女、儿童、老龄等工作。

三、加强政府自身建设

按照党中央、国务院，省委、省政府和市委的部署要求，顺应时代发展的新需要和人民群众的新期待，努力建设为民务实清廉高效政府。

（一）切实转变政府职能。抓实各项改革举措，处理好政府和市场的关系，切实简政放权，把政府该放的权放开放到位，使市场在资源配置中起决定性作用；把政府该管的事管好管住，切实履好市场监管、社会管理和公共服务职责，切实把精力和重点转到为企业提供优质服务、为百姓营造安居乐业的环境上来，切实想大事、干实事、解难事。巩固和深化“两集中两到位”改革，进一步精简审批事项，优化审批流程，提高审批效能，不让企业来回奔波耽误时间和精力，不让办事群众“求爷爷拜奶奶”，真正把政务中心打造成“政务超市”，让政务窗口成为办事窗口。

（二）切实加强依法行政。深入推进法治政府建设，严格实行“三重一大”集体决策等制度，规范管人、管事、管钱。认真落实市人大及其常委会的决议决定，主动接受人大法律监督和工作监督、政协民主监督，高效办理人大议案和政协提案。推进政务公开，明确每项行政许可的办理条件、办理程序、办理时限，接受社会监督，确保权力阳光运行。推行刚性科学预算，杜绝“零批碎要”、“人情增支”现象。严格按照法定权限和程序履职，严格行政处罚裁量权基准，坚决杜绝行政不作为、缓作为和乱作为，做到政府职权法授、程序法定、行为法限、责任法究。

（三）切实改进工作作风。扎实开展党的群众路线教育实践活动，严格执行中央“八项规定”和省委“九条规定”，坚决克服形式主义、官僚主义、享乐主义和奢靡之风，做到令行禁止，确保政令畅通，把全部心思和精力投入到谋发展、抓落实上。少讲官话，多接地气；少坐机关，多下基层；少听汇报，多查实情，实地感受群众疾苦，上门为企业排忧解难。将心比心，以心换心，把企业当朋友，把百姓当亲人，以政府的勤奋和辛劳，切实增强娄底发展保障和群众幸福感觉。

（四）切实加强廉政建设。推行权力清单制度，把权力关进制度的笼子里。深入开展超标配备公车及公车私用、境外招商奢侈浪费和高消费问题、公款送礼、“庸懒散”、违规职务消费和公款大吃大喝问题等五个专项整治工作，坚决执行厉行节约、反对浪费的有关规定，从严从紧控制行政成本，全面实行公务卡、公务接待集中管理和审批控制制度，坚决做到办公性行政经费零增长、“三公”经费只减不增，全面停止新建机关楼堂馆所。加强反腐倡廉建设，严格落实“一岗双责”。加强审计监督和行政监察，坚决查处违法、违规、违纪的人和事，以务实清廉形象，打造人民信任和满意的政府！

各位代表、同志们，真抓实干成就伟业，奋力拼搏创造辉煌。站在新的起点，我们必须牢记昨天的艰辛、无愧今天的使命、不负明天的梦想。让我们在市委的坚强领导下，凝心聚力，奋力开拓，不断开创“科学发展、加速赶超”新局面，为加快建设幸福娄底、全面建成小康社会而努力奋斗！
